# Silver Nanoparticles as an Intracanal Medicament: A Scoping Review

**DOI:** 10.1155/2023/9451685

**Published:** 2023-09-11

**Authors:** Azra Kaukab, Sumit Gaur, Rupali Agnihotri, Vani Taneja

**Affiliations:** ^1^Department of Pedodontics and Preventive Dentistry, Manipal College of Dental Sciences, Manipal Academy of Higher Education (MAHE), Manipal 576104, Karnataka, India; ^2^Department of Periodontology, Manipal College of Dental Sciences, Manipal Academy of Higher Education (MAHE), Manipal 576104, Karnataka, India; ^3^Department of Pediatric Dentistry, Dentistry Program, Batterjee Medical College, Jeddah 21442, Saudi Arabia

## Abstract

Silver nanoparticles (AgNPs) release Ag ions with potent bactericidal and anti-inflammatory effects. They have shown promising results as an intracanal medicament for removing *Enterococcus faecalis* (*E. faecalis*), a resistant bacterium associated with root canal failures. This review summarizes the role of AgNPs as an intracanal medicament. Original research articles on AgNPs as an intracanal medicament were searched in databases such as MEDLINE (PubMed), Scopus, and Embase, resulting in 24 studies. They showed that AgNPs effectively eliminated *E. faecalis* and reduced postoperative pain following root canal therapy. However, these effects should be further verified through clinical trials as most of the studies were in vitro.

## 1. Introduction

Root canal disease, a biofilm-mediated infection, is successfully managed through the reduction or elimination of the biofilm by adequate debridement, disinfection, and obturation with an excellent apical seal [1].

Resistant periradicular lesions are predominantly associated with *Enterococcus faecalis* (*E. faecalis*) and are often detected in the monocultures of the root canals of teeth [2]. It is a facultative anaerobic Gram-positive microorganism strongly associated with secondary endodontic infections and failures. In addition, *Fusobacterium nucleatum* (*F. nucleatum*) is frequently associated with immature necrotic teeth [3], and *Candida albicans* (*C. albicans*) has been extracted from infected root canals along with *E. faecalis* [4].

*E. faecalis* can withstand extreme conditions due to its characteristic endurance to antibiotics, irrigant solutions, intracanal medicaments, and high alkaline pH [5]. Besides, it forms biofilms that penetrate the dentinal tubules, complicating its elimination from root canals [6]. The biomechanical preparation and shaping of the root canal efficiently reduce the number of microorganisms in the root canals but cannot remove them from the isthmuses, lateral canals, and apical deltas [7].

Calcium hydroxide (Ca(OH)_2_) is a widely applied intracanal medicament between appointments to reduce canal bacteria. It is antibacterial, and during apexogenesis, or pulp capping, it stimulates hard tissue formation, dissolves the tissues, and promotes apical exudate elimination [8]. It produces nonspecific bactericidal action due to high alkalinity, as most microorganisms are eliminated at a pH of 9.5–12, and only a few survive at a pH of 11 or greater [9]. Despite these antimicrobial properties, Ca(OH)_2_ is ineffective against polymicrobial infections or bacterial biofilms. However, its combination with antimicrobials such as chlorhexidine digluconate (CHX) has shown synergistic antibacterial action [10].

Lately, nanotechnology in dentistry has promoted the development of excellent biomaterials with unique physical, chemical, and biological properties [11]. The nanoparticles (NPs) have superior antibacterial effects due to their higher volume-to-surface area ratio and lesser particle dimension, which results in higher effective contact and larger reaction surface. Subsequently, they penetrate the dentinal tubules and produce a prolonged antibacterial impact at the infection site at reduced doses [12].

Among the various NPs, silver nanoparticles (AgNPs) are broad-spectrum and biocompatible, with sizes ranging from 1 to 100 nm [13, 14]. They release Ag ions, producing robust bactericidal effects against Gram-positive, Gram-negative, and multidrug-resistant bacteria [15, 16]. Although Ag is an inert material in the bulk state, it gets ionized by moisture and converts into a highly reactive state [17]. AgNPs exert bactericidal action by destroying the cell envelope, inhibiting metabolic enzymes, and generating reactive oxygen species (ROS) [18]. Moreover, they gather in the pits on the cell surface and denature the cell membrane leading to cell lysis and death. AgNPs are used as an intracanal medicament with an appropriate vehicle. Studies show their increased effectiveness compared against Ca(OH)_2_ alone in removing *E. faecalis* [1, 10], while a few evaluated their effects against other agents [4, 19].

With this background, the present review summarizes the role of AgNPs as an intracanal medicament.

## 2. Materials and Methods

**Figure 1:**
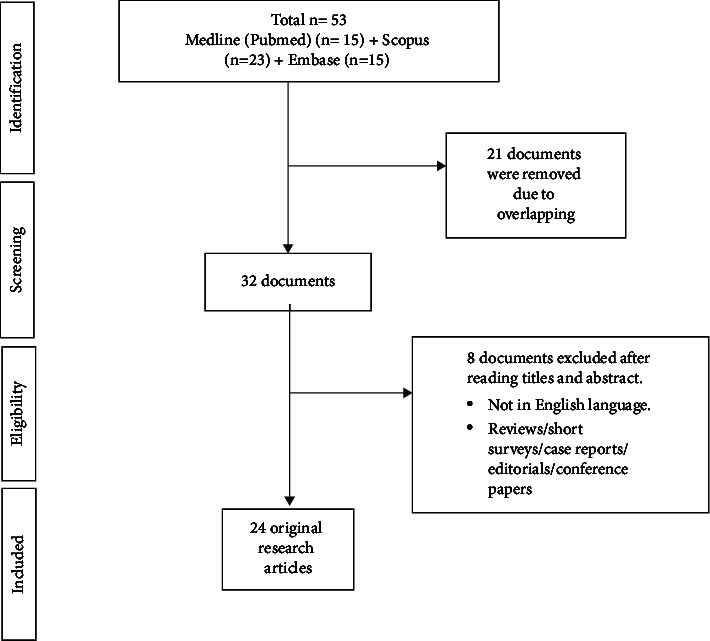
Evidence search for the role of AgNPs as an intracanal medicament.

Databases such as Scopus, Embase, and MEDLINE (PubMed) were searched according to the Preferred Reporting Items for Systematic Reviews and Meta-Analyses extension for Scoping Reviews (PRISMA-ScR) guidelines to identify research publications on the application of AgNPs as intracanal medicaments. The keywords applied during the initial search were “silver” AND “nanoparticles” AND “intracanal” AND “medicament,” resulting in 53 articles, of which the titles and abstracts of 32 articles were read, leading to 24 articles that were included in the review [1, 3, 4, 9, 10, 12, 19–36] (Figure 1).

### 2.1. Inclusion and Exclusion Criteria

Original research full-text articles in English on the application of AgNPs as an intracanal medicament were included. Conference proceedings, recommendations, expert statements, technical reports, reviews, case reports, and nonoriginal papers were excluded.

### 2.2. Risk of Bias Assessment

The ROB-2 and Quin tools were used to assess the risk of bias in randomized controlled clinical trials (RCTs) and in vitro studies, respectively [37, 38]. The ROB-2 tool has six criteria, including selection, performance, detection, attrition, and reporting bias, and showed a low risk of bias in both the included RCTs. The Quin tool applies 12 criteria, of which only nine were considered relevant for the included studies. They were statements on aims and objectives, sample size calculation, sampling technique explanation, comparison group details, details on randomization, outcome measurements, statistical analysis, and presentation of results. The criteria were scored, and the study was graded as high, medium, or low risk (Table 1). There was a low-to-medium risk of bias in the included in vitro studies. Two authors (SG and RA) did the risk of bias assessment, and disagreements were resolved by discussion among all the authors.

### 2.3. Data Extraction

The aims and objectives of the study, the type of study, AgNPs' form and concentration, the antimicrobial effect, results, and conclusions were recorded (Table 2).

## 3. Results

Of the 24 included studies, nineteen were in vitro [1, 9, 10, 12, 19–23, 26–29, 31–36], three ex vivo [3, 4, 25], and two in vivo [24, 30]. About 16 studies evaluated the antibacterial efficacy of AgNPs as an intracanal medicament [1, 3, 4, 9, 10, 19, 21–30]. Two studies analyzed the crown discoloration caused by AgNPs [20, 36], three reported the methods of their synthesis [12, 31, 32], and three reported recent advances in AgNPs as intracanal medicaments [33–35].

### 3.1. Synthesis of AgNPs

The studies applied both synthetic and biosynthetic AgNPs. The biosynthesized AgNPs were synthesized from an endophytic fungus, *Fusarium semitectum*, isolated from the fresh leaves of *Withania somnifera* (Ashwagandha) [12]. Likewise, fresh leaves of *Andrographis paniculata* (*A. paniculata*) and *Ocimum sanctum Linn* (*O. sanctum Linn*) were utilized for the green synthesis of AgNPs [31]. They were also synthesized by using silver nitrate (precursor salt), water (solvent), maltose (reducing agent), and gelatin (stabilizer), eliminating the need for toxic, nonbiodegradable chemicals [32].

### 3.2. Combinations of AgNPs

AgNPs were either evaluated alone or in combination with other intracanal medicaments such as Ca(OH)_2_ or 2% CHX in a ratio of 1 : 1 or AgNPs with aloe vera gel [1, 4, 10, 23]. The AgNP suspension had a mean particle size of 2 nm, 8–12 nm, 20 nm, 25–40 nm, and 70 nm [1, 4, 9, 10, 19, 22, 24, 27, 28, 30] with a concentration of 20 ppm and 100–200 ppm [1, 4, 9, 10, 22, 30]. The suspension was mixed with Ca(OH)_2_ in a ratio of 1 : 1 or 1 : 2 [1, 4, 9, 10, 28]. In gel preparations, AgNP concentrations ranging from 0.01% to 0.15%, 0.02%, and 1.2% were effective against *E. faecalis*, while a 0.06% concentration was effective against *F. nucleatum* [3, 25, 27, 28].

### 3.3. Antimicrobial Efficacy

The positively charged AgNPs, with a large surface area, interact with the negatively charged cell membrane and exert an antimicrobial effect. They interact with the bacterial membrane's “building elements” leading to changes in structure and degradation and, finally, cell lysis [39]. They get internalized and generate ROS, which damage intracellular macromolecules such as DNA and proteins.

The studies assessed the antimicrobial efficacy of AgNPs against *E. faecalis* [1, 4, 9, 10, 12, 19, 21–23, 25–31, 34], *C. albicans* [4, 31], *F. nucleatum* [3], *Streptococcus mutans* (*S. mutans*), *Staphylococcus aureus* (*S. aureus*), and *Pseudomonas* [31].

When the antimicrobial effect of various concentrations of AgNPs was evaluated, it was found that concentrations superior or equal to 300 *μ*g/mL decreased *E. faecalis* growth by more than 90%, i.e., it was the minimum inhibitory concentration (MIC) for the bacteria. However, no growth was observed from 900 *μ*g/mL onward, indicating that it was the minimum bactericidal concentration with the bacteriostatic effect at 300 *μ*g/mL [27]. Moreover, their antimicrobial effects at 300 *μ*g/ml and 500 *μ*g/ml were equivalent to the action of Ca(OH)_2_ [27]. Likewise, other concentrations of AgNPs ranged between 0.12 and 17 *μ*g/ml of which 1.05 *μ*g/ml was the MIC for *E. faecalis* [19]. Besides, the AgNPs of aloe vera inhibited *E. faecalis*, *S. mutans*, and *C. albicans* in the concentration order 2000 *μ*g/ml > 1000 *μ*g/ml > 500 *μ*g/ml [23]. When various concentrations of AgNPs (0.03%, 0.04%, and 0.06%) were evaluated for their efficacy against *F. nucleatum*, they reduced the bacterial count in the increasing order of their concentration, with 0.04% of AgNPs being the lowest concentration effective against *F. nucleatum* [3].

Combination of Ca(OH)_2_ with AgNPs was more efficient than Ca(OH)_2_ alone or other intracanal medicaments like CHX for reducing *E. faecalis* [1, 10, 21, 22, 28] and *F. nucleatum* [3]. The antibiofilm effect of Ca(OH)_2_ with AgNPs (0.02%) [25, 28] was not significantly different from 1 mg/ml of triple antibiotic paste [28]. Some studies showed similar antibacterial effects of AgNPs, Ca(OH)_2_, and nano-Ca(OH)_2_ [27, 30, 31] or AgNPs and 2% CHX functionalized nanoparticles when used separately [19, 25]. However, AgNPs (100 ppm) combined with Ca(OH)_2_ and CHX showed the most significant reduction of *E. faecalis* [1, 10, 26, 29]. Although their 1 : 1 ratio [1, 10] significantly reduced the *E. faecalis* counts during the first week, no significant difference was reported after one month [1]. Moreover, no significant difference was observed in the antimicrobial activity of Ca(OH)_2_ and Ca(OH)_2_ with AgNPs against *S. mutans*, *E. faecalis*, and *Pseudomonas* at all tested concentrations. The mean zone of inhibition was greater for the combination of Ca(OH)_2_ and AgNPs compared to Ca(OH)_2_ at all tested concentrations against *S. aureus* and *C. albicans* although it was not statistically significant for *S. aureus* [31].

Various concentrations of AgNPs (0.03%, 0.04%, and 0.06%) with Ca (OH)_2_ reduced the *F. nucleatum* colonies at 7 and 14 days. Combining 0.06% AgNPs with Ca (OH)_2_ was most effective against *F. nucleatum*, and their efficacy increased with time [3].

However, a study showed the most significant reduction in the bacterial count with Ca(OH)_2_ alone, followed by AgNPs combination with Ca(OH)_2_ (1 : 2) and AgNPs alone during the first and second weeks of exposure to the intracanal medicament [9]. Moreover, another study showed that 2% CHX was most effective against *E. faecalis* and *C. albicans*, followed by AgNPs' combination with either 2% CHX and Ca(OH)_2_ or AgNPs alone [4].

### 3.4. Other Effects

AgNPs produced a more significant reduction of initial postoperative pain than Ca(OH)_2_ alone, as indicated in the clinical trials on subjects undergoing endodontic treatment [24, 30].

### 3.5. Adverse Effects

Even though the Ag ions may cause gray-black discoloration of a crown over time, the AgNPs combined with Ca(OH)_2_ caused no substantial tooth discoloration at one-week, one-month, and three-month intervals [20]. Similarly, a comparison of crown discoloration after applying AgNPs and Ca(OH)_2_ or AgNPs and graphene oxide showed no significant discoloration at 15 days. However, it was noted at the one-month interval [36].

### 3.6. Recent Advances

Some studies utilized mesoporous calcium-silicate NPs loaded with low-dose Ag ions, and Triton X-100 was used for the controlled delivery of AgNPs (M-AgTx). These particles inactivated a 28-day *E. faecalis* biofilm [33]. Likewise, M-AgTx showed excellent antibacterial ability against *E. faecalis* and high substantivity on dentin [34]. AgNPs with graphene oxide had an almost negligible effect on the root dentin microhardness compared to Ca(OH)_2_ alone or when combined with AgNPs [35].

## 4. Discussion

The included studies show that AgNPs as intracanal medicaments have potent antibacterial effects against *E. faecalis*, *F. nucleatum*, and *C. albicans*, comparable to Ca(OH)_2_ and CHX.

A few studies revealed that green biosynthesis using plants and fungi developed AgNPs that were equally effective against *E. faecalis* [12, 31, 32], reducing costs and the risk of toxic byproducts. AgNPs biosynthesized from fungi showed antimicrobial efficacy similar to 2% CHX. Fungi require simple nutrients for growth, a cost-effective and easy process involved in the synthesis of AgNPs [12]. They are like “nanofactories,” producing particles with good monodispersity, size, and chemical composition. Likewise, plants such as *A. paniculata* and *O. sanctum Linn* were also applied for their synthesis [31]. The active compound in *A. paniculata* is andrographolide, which inhibits quorum-sensing and microbial virulence factors. At the same time, *O. sanctum Linn* produces camphor, eucalyptol, eugenol, alpha-bisabolene, beta-bisabolene, and beta-caryophyllene responsible for its antimicrobial action. They were effective against *S. aureus*, *C. albicans*, and *E. faecalis* [31]. These plants contain crude metabolites, including phenolic acid, flavonoids, alkaloids, and terpenoids, which act as reducing agents to generate AgNPs from silver nitrate solution. No reports show whether biogenic NPs synthesized from various sources, including fungi or plants, are superior or inferior in their activities. However, those derived from fungi may be beneficial in manufacturing owing to the more significant amounts of metabolites produced. Besides, fungi produce antibiotics which act in synergy with the NPs [40].

AgNPs, when combined with Ca(OH)_2_, reduced the colony-forming units (CFUs) of *E. faecalis* within one week of the application when compared to Ca(OH)_2_ alone [1, 10, 22]. It was suggested that adding AgNPs to Ca(OH)_2_ reduced its MIC against *E. faecalis* and increased the antimicrobial efficacy of the medicament [41]. Moreover, AgNPs in gel form were used as a vehicle for Ca(OH)_2_, which ensured a prolonged interaction with bacterial cell walls, disrupted bacterial integrity, and reduced the CFUs of *E. faecalis* [42]. Besides Ca(OH)_2_ and CHX, triple and dual antibiotic pastes were compared with AgNPs [10]. CHX (2%) has substantivity properties and an antimicrobial effect due to its positively charged molecules, which are adsorbed into the dentin and persist there for an extended duration, which prevents bacterial colonization. The triple and dual antibiotic pastes showed more significant inhibition of *E. faecalis* but are associated with bacterial resistance, tooth discoloration, and changes in the dentin, leading to increased demineralization and fracture [10]. However, AgNPs have a reduced risk of bacterial resistance due to the following four well-defined antimicrobial mechanisms [18, 27]: firstly, they adhere to the cell wall and membrane surface; secondly, they penetrate the cell and damage intracellular structures such as mitochondria, vacuoles, ribosomes, and biomolecules, including proteins, lipids, and DNA; thirdly, they induce cellular toxicity and oxidative stress by generating ROS and free radicals; and fourthly, they modulate signal transduction pathways. Besides, they also modulate the immune system of the human cells and influence the inflammatory response, further inhibiting microorganisms [18]. These modes of action reduce the postoperative pain for up to 24 hours following root canal treatment [30].

Although AgNPs have less probability of developing bacterial resistance, lately, some studies have reported that certain endodontic bacteria may develop resistance to them by various mechanisms [43]. They include intrinsic (such as efflux pumps, porins' downregulation, and chromosomal resistance genes) or extrinsic (such as point and adaptive mutations and plasmids with resistance genes) adaptation systems [43]. AgNPs activate the envelope stress response, including positive charges into the bacterial cell wall. Subsequently, the bacteria equalize the electrical charge with the surface of AgNPs, leading to repulsion. Moreover, AgNP concentrations below the MIC lead to oxidative stress and the appearance of persistent bacteria such as *E. faecalis*. They develop cell dormancy, wherein the antibiotic binds to the bacteria but cannot kill them due to the downstream pathways' inactivation. The nonlethal concentrations of AgNPs increase bacterial mutation due to ROS production and DNA damage. These mutations upregulate efflux pump genes and downregulate porins, causing a resistant phenotype in bacteria. The extrachromosomal genetic elements like plasmids enter the bacterial cells through active and passive mechanisms and promote lateral transfer of genes resistant to AgNPs.

Moreover, increased AgNP usage may stimulate coresistance and coregulation to metals and antibiotics in bacteria. Therefore, they would develop antibiotic resistance when exposed to nonbactericidal concentrations of AgNPs. Lastly, the sublethal exposure to AgNPs may enhance biofilm development, upregulate lipopolysaccharide formation, gene transfer, and efflux pump genes, enhance the protein and sugar levels in the biofilm, and promote the development of more effective resistance mechanisms against the antimicrobial effect of AgNPs [43].

However, recent advances in AgNPs, like M-AgTx, may overcome these bacterial resistance mechanisms by coreleasing Ag^+^ and TX-100, which eliminate bacteria in a concentration- and time-dependent manner [33]. TX-100 increased the cell permeability and promoted the penetration of M-AgTx into the bacterial cell leading to cell perforation and breakdown. This mechanism of TX-100 decreased the alkaline and Ag^+^ resistance of *E. faecalis* and enhanced the antibacterial effect of silver and hydroxide ions of M-AgTx against *E. faecalis*. Furthermore, combinations of graphene oxide with AgNPs improved the microhardness of treated dentine due to AgNPs' deposition inside the dentinal tubules [35]. A study showed tooth discoloration with AgNPs, after their application as an intracanal medicament for a month [36]. Usually, imidazolium-coated AgNPs produce staining like blood [44]. The discoloration is mainly due to dentinal tubule infiltration. Therefore, their application should be limited to the root canal and not extend to the pulp chamber. Any medicament remains should be eliminated before the crown placement [44, 45].

The biodistribution and toxicity studies in rats and mice showed that AgNPs administered by inhalation, ingestion, or intravenous or intraperitoneal injections are found in blood and are toxic to the lungs, liver, kidneys, intestines, and brain [46].

The toxicity is positively associated with free silver ion levels and is related to their nanosize, interrupting the bioactive molecules, eukaryotic cells, and tissues [45]. Their physical and chemical structures affect the cell response. The oxidative stress of AgNPs toxicity produces free radicals, accumulating in the cell nucleus and cytoplasm. Due to their large surface area, AgNPs produce significant early toxicity, which decreases with time with their interaction with the organic compounds.

Moreover, they accumulate in the liver and spleen when used in high doses. They can also penetrate the blood-brain barrier by transsynaptic transport and accumulate in the brain [45, 47]. Nevertheless, any levels of accumulated silver are cleared from the body by eight weeks, although disproportionately high levels of AgNPs destroy the mitochondrial function. When applied in concentrations greater than 200 mg/kg body weight, they generate free radicals, release ROS, and cause cell damage. However, various animal models have not shown the toxic effects of AgNPs with an average size of 5 nm and concentration of 25 g/mL or when administered orally [45]. A commercially available AgNPs colloid did not alter metabolism, blood, urine, vital signs, or physical or radiographic findings and was excreted in feces, with only a minuscule amount absorbed [45]. However, AgNP toxicology research on susceptible individuals and human exposure is understudied. There is a lack of detailed research on the effects of AgNPs on humans via various routes of exposure. Few studies evaluated whether AgNPs penetrated the physically and functionally intact human skin [48, 49]. They demonstrated that AgNPs (10–40 nm) penetrated the normal skin of healthy human participants [48]. When applied as a nanocrystalline silver dressing for four to six days, AgNPs penetrated beyond the stratum corneum and reached the reticular dermis. Besides, an oral dosage of 10 ppm of AgNPs (5–10 nm) produced no physical metabolic, hematologic, urine, or imaging morphology changes in humans [50].

Although the included studies support the antibacterial effects of AgNPs against intracanal pathogens like *E. faecalis*, most of the results were derived from in vitro investigations. The antibacterial properties of AgNPs depend on their type, formation method applied, and concentration. Moreover, their low dose was effective as an intracanal medicament, specifically *E. faecalis*. However, caution should be exercised as significant usage of AgNPs in sublethal concentrations may lead to bacterial resistance, which was not evaluated in the included studies. Further studies are necessary to evaluate the ideal concentration of AgNPs for favorable antimicrobial effects without inducing cytotoxicity in vivo. As AgNPs produce synergistic antibacterial effects when combined with commonly used medicaments such as Ca(OH)_2_ and CHX, newer NP-based formulations should be produced for successful root canal therapy. Besides, their tooth discoloration potential should be investigated.

## 5. Other Applications of AgNPs

AgNPs have various medicinal uses such as antimicrobials, antiviral, nematicidal, anthelmintic, anticancer, bone healing, wound repair promoters, vaccine adjuvants, antidiabetic agents, and biosensors as explained in an elaborate review of AgNPs by Xu et al. [51].

### 5.1. Antimicrobial, Antiviral, Nematicidal, and Anthelmintic Activity

AgNPs inhibit pathogenic bacteria, fungi, and viruses, including *Staphylococcus aureus*, *Escherichia coli*, *Pseudomonas aeruginosa*, dermatophyte, and HIV-1. As they are broad-spectrum antibacterials, they are applied in catheter modification, wound healing, and bone healing [51]. In addition, they are antiviral against *hepatitis B virus* (*HBV*) [52], *human parainfluenza virus* (*HPIV*), *herpes simplex virus* (*HSV*) [53], and *influenza A* (*H1N1*) *virus* [54]. AgNPs are helpful antivirals due to their reduced size (<10 nm), which enhances their large surface area and ability to adhere to the virus surface. They bind to the glycoprotein knobs, inhibit the reverse transcriptase enzyme, and interact with the virus in size- and dose-dependent manner [55]. They either block the virus's contact with cells or directly inactivate it. Furthermore, they alter the cellular targets responsible for drug resistance and pathogenicity in resistant fungi. In *C. albicans*, AgNPs act on oleic acid targets that are significant for hyphal morphogenesis and pathogenicity. They saturate, adhere to the fungal hypha, and inactivate the fungus [51, 56].

AgNPs are also effective nematicidal and anthelmintic and kill filaria and larvae [51]. They induce cell apoptosis and destroy them mainly through ROS generation. Specifically, AgNPs synthesized from *Acacia auriculiformis* effectively killed filaria [51, 57]. Furthermore, AgNPs are active against *Haemonchus contortus* [58], larvae of *Anopheles stephensi*, and *Culex quinquefasciatus*, which may prevent malaria and filariasis [51, 59].

### 5.2. Anticancer Effect

AgNPs produce a wide range of anticancer effects as they influence cancer cells' proliferation, viability, apoptosis, and necrosis by destroying their ultrastructure, inducing ROS production, and DNA damage [51]. AgNPs promote apoptosis by regulating the expression of vital genes like p53 that regulate essential signaling pathways, such as the hypoxia-inducible factor pathway. It induces sub-G1 cell cycle arrest and apoptosis in several cancer cells exposed to AgNPs [51, 60]. They also prevent metastasis by impeding tumor cell migration and angiogenesis [51, 61].

### 5.3. Wound Repair and Bone Healing

AgNPs increase the wound healing rate and produce notable cosmetic results with near-average hair growth and reduced hypertrophic scarring. AgNPs reduce TGF-*β* levels, increase interferon-*γ* and vascular endothelial growth factor mRNA in keratinocytes at the wound edge, and promote wound healing by inducing angiogenesis [51]. They persist in the fibroblast cytoplasm and stimulate dermis and epidermis restoration. They induce the proliferation and migration of keratinocytes, reduce collagen and hydroxyproline levels, and promote fibroblast differentiation into myofibroblasts, leading to early wound adhesion, contraction, and closure [51, 62].

Furthermore, AgNP-implanted crystallized hydroxyapatite or titanium scaffolds have an antibacterial effect on bone defects [51, 63]. As they are osteoconductive, they stimulate mesenchymal stem cell proliferation and osteogenic differentiation during fracture healing [51].

### 5.4. Vaccine Adjuvant

The vaccine adjuvants reduce the amount of antigen required, shorten the time required for a protective threshold of antibody production, improve the intensity of the elicited responses, and stimulate long-term memory responses to reduce the requirement of repeated vaccinations. AgNPs are immunological adjuvants as they stimulate Th2-biased immune responses through increased serum antigen-specific IgG and IgE production and activate and recruit local leukocytes and macrophages [51].

### 5.5. Antidiabetic Agent

AgNPs derived from plant extracts are antidiabetic. For example, AgNPs synthesized from the *Solanum nigrum* leaf extract reduced the blood glucose levels in diabetic rats [51, 64]. Compared to glibenclamide, they produced an excellent hypoglycemic effect. Similarly, AgNPs synthesized from the *Argyreia nervosa* leaf extract inhibited *α*-amylase and *α*-glucosidase, producing an antidiabetic effect [51, 65]. AgNPs influence insulin signaling or sensitivity by activating protein kinase C and PI3K pathways at the insulin receptor substrate level. They inhibit protein kinase C isozyme, enhance insulin sensitivity and secretion, and reduce insulin resistance [51].

### 5.6. Biosensor and Imaging

AgNPs are a cost-effective surface-enhanced Raman scattering substrate (SERS) [51]. They are applied as biosensors to identify blood glucose, enzymes, tumor markers, and pathogens. The nanostructure and large surface area of carriers enhance the interaction between AgNPs and electrodes and accelerate the electron transfer of AgNPs, leading to improved biosensor sensitivity [51, 66]. For instance, AgNPs combined with graphene oxide NPs enabled SERS biosensing and drug delivery [51, 67]. They are also used as synthetic probes to detect mercury content in water, soil, and food, detect copper ions in blood samples, and detect sickle cell anemia mutations [51].

## 6. Conclusion

This review shows that AgNPs have antibacterial efficacy comparable to Ca(OH)_2_, and this effect increases when the two materials are combined and used as intracanal medicaments. However, further in vivo clinical trials are needed to determine the effective AgNP antibacterial concentrations and plausible adverse effects.

## Figures and Tables

**Table 1:** Risk of bias assessment of in vitro studies and randomized controlled clinical trials.

In vitro studies (Quin tool)																			
Criteria	Bednarski et al. [21]	Javidi et al. [22]	Afkhami et al. [1]	Venkateshbabu et al. [19]	Fan et al. [34]	Saad and Alabdulmohsen et al. [9]	Afkhami et al. [20]	Haripriya and Ajitha [23]	Halkai et al. [12]	Jhamb et al. [26]	Jaju and Nasim [36]	Marín-Correa et al. [27]	Balto et al. [28]	Bruniera et al. [32]	Sethi et al. [29]	Duan et al. [33]	Afkhami et al. [10]	Nasim et al. [31]	Nasim et al. [35]
Clearly stated aims/objectives	2	2	2	2	2	2	2	2	2	2	2	2	2	2	2	2	2	2	2
Detailed explanation of sample size calculation	0	0	0	0	Not applicable	0	0	Not applicable	Not applicable	0	0	0	2	Not applicable	0	0	0	Not applicable	0
Detailed explanation of sampling technique	2	2	2	2	2	2	2	0	0	0	2	2	2	Not applicable	0	2	2	2	2
Details of comparison group	2	2	2	2	2	2	2	2	2	2	2	2	2	Not applicable	2	2	2	2	2
Detailed explanation of methodology	2	2	2	2	2	2	2	2	2	2	2	2	2	Not applicable	2	2	2	2	2
Randomization	1	1	1	1	Not applicable	1	1	Not applicable	Not applicable	0	1	0	1	Not applicable	0	0	1	Not applicable	1
Method of measurement of outcome	2	2	2	2	2	2	2	2	2	2	2	2	2	2	2	2	2	2	2
Statistical analysis	2	2	2	2	2	2	2	2	2	2	2	2	2	2	2	2	2	2	2
Presentation of results	2	2	2	2	2	2	2	2	2	2	2	2	2	2	2	2	2	2	2
Total score	15	15	15	15	14	15	15	12	12	12	15	14	17	8	12	14	15	14	15
Risk of bias	Low	Low	Low	Low	Low	Low	Low	Low	Low	Medium	Low	Low	Low	Low	Medium	Low	Low	Low	Low

Randomized controlled clinical trials (ROB2 tool)																			
Bias domain						El Abbasy et al. [24]							Fahim et al. [30]						

Selection bias			Random sequence generation			✓							✓						
			Allocation concealment			✓							✓						
Performance bias						✓							✓						
Detection bias						✓							✓						
Attrition bias						✓							✓						
Reporting bias						✓							✓						
Other bias						NA							NA						
Overall risk of bias						Low							Low						

**Table 2:** Studies reporting antimicrobial effect of AgNPs as an intracanal medicament.

Author	Conclusion
Bednarski et al. [21]	(i) Zero colony-forming units (CFUs) with nanocare with AgNPs
Javidi et al. [22]	(ii) The CFUs observed after Ca(OH)_2_ with AgNPs dressing were significantly less than those observed with Ca(OH)_2_ alone
Afkhami et al. [1]	(iii) Reduced *E. faecalis* colonies in AgNPs with Ca(OH)_2_ group
Venkateshbabu et al. [19]	(iv) Effective dentin disinfection from *E. faecalis* with AgNPs
Saad and Alabdulmohsen [9]	(v) Antibacterial effect of AgNPs was lower than that of Ca(OH)_2_ or the combination of both materials
Yadav et al. [4]	(vi) 2% chlorhexidine was more effective as an intracanal medicament when compared to AgNPs and combination of AgNPs with Ca(OH)_2_ or chlorhexidine against *E. faecalis*
Haripriya and Ajitha [23]	(vii) Ag-NPs of aloe vera had antimicrobial effect
El Abbasy et al. [24]	(viii) Reduced postoperative pain in the AgNPs group compared to the Ca(OH)_2_ group
Heidar et al. [25]	(ix) Effective antibacterial activity of AgNPs against *E. faecalis* biofilm
Jhamb et al. [26]	(x) Combination of silver nanocure gel (AgNPs), Cavisept (chlorhexidine gel), and Aveu-Cal gel showed the greatest antibacterial effect
Marín-Correa et al. [27]	(xi) Antimicrobial effect of AgNPs against *E. faecalis* is equivalent to Ca(OH)_2_
Balto et al. [28]	(xii) Antibacterial effect of AgNPs with Ca(OH)_2_ was comparable to triple antibiotic paste
Sethi et al. [29]	(xiii) Combination of silver nanocure gel (AgNPs), Cavisept (chlorhexidine gel), and Aveu-Cal gel showed the greatest antibacterial effect
Afkhami et al. [10]	(xiv) Zero colony count with double and triple antibiotic pastes followed by Ca(OH)_2_ with AgNPs
AlGazlan et al. [3]	(xv) Combination of AgNPs with Ca(OH)_2_ was more effective against *F. nucleatum*
Fahim et al. [30]	(xvi) Antibacterial effect of the AgNPs and nano-Ca(OH)_2_ was equivalent to that of Ca(OH)_2_ but produced better pain control

## Data Availability

All the data used to support the findings of this review are included within the article.
